# Supplementary material for: Adaptive expansion of ERVK solo-LTRs is associated with Passeriformes speciation events
Source: Nat Commun. 2024 Apr 11;15:3151. doi: 10.1038/s41467-024-47501-3 (PMC11009239; doi:10.1038/s41467-024-47501-3)
Supplement: Supplementary file 1 — Supplementary Information [file 41467_2024_47501_MOESM1_ESM.pdf]

Supplementary information for “Adaptive expansion of ERVK solo-LTRs is associated with Passeriformes speciation events”

Guangji Chen[1,2,3], Dan Yu[2,4], Yu Yang[5], Xiang Li[6], Xiaojing Wang[6], Danyang Sun[2,4], Yanlin Lu[2,4], Rongqin Ke[5], Guojie Zhang[2,7], Jie Cui[8,9,10,11,*], Shaohong Feng[2,7,12,*]

1. College of Life Sciences, University of Chinese Academy of Sciences, Beijing, China
2. Center for Evolutionary & Organismal Biology, Zhejiang University School of Medicine, Hangzhou, China
3. BGI Research, Wuhan, China
4. Center for Genomic Research, International Institutes of Medicine, The Fourth Affiliated Hospital, Zhejiang University School of Medicine, Yiwu, Zhejiang, China
5. School of Medicine, Huaqiao University, Xiamen, Fujian 361021, China
6. CAS Key Laboratory of Molecular Virology & Immunology, Shanghai Institute of Immunity and Infection, Chinese Academy of Sciences, Shanghai, China
7. Liangzhu Laboratory, Zhejiang University, Hangzhou 311121, China
8. Department of Infectious Diseases, Shanghai Key Laboratory of Infectious Diseases and Biosafety Emergency Response, National Medical Center for Infectious Diseases, Huashan Hospital, Shanghai Medical College, Fudan University, Shanghai, China
9. Laboratory for Marine Biology and Biotechnology, Qingdao Marine Science and Technology Center, Qingdao, China.
10. Shanghai Huashen Institute of Microbes and Infections, Shanghai, China
11. The Institute of Infection and Health Research, Fudan University, Shanghai, China
12. Department of General Surgery of Sir Run Run Shaw Hospital, Zhejiang University School of Medicine, Hangzhou, China

*Correspondence to: Jie Cui (jiecui@fudan.edu.cn) and Shaohong Feng (fengshaohong@zju.edu.cn).

Supplementary Note

Threshold test for solo-LTR identification

As the quality of genome assemblies could affect the identification of solo-LTRs, we evaluated the effect on the solo-LTR identification after removing the scaffold length of less than 10 kb, 15 kb, and 20 kb (**Supplementary Fig. 10**). To distinguish solo-LTRs from paired-LTRs, we tested two thresholds of nucleotide identity (75% and 85%), which approximated the threshold of 80% identity used to classify TEs into the same family[1]. No significant differences were found among the results obtained from the application of different filtering criteria (**Supplementary Data 7**). Therefore, we applied more stringent filtering criteria, by using scaffolds greater than 20 kb and similarities greater than 85% to

identify solo-LTRs. Finally, we annotated all detected solo-LTRs using RepeatMasker (v4.1.2) with the RepBase library (v.20170127) to identify the sources of solo-LTRs among mammals, reptiles, and birds.

Evolutionary patterns of solo-LTRs with the target site duplications

Following Peona et al.[2], we also examined the target site duplications (TSDs) at the extremities of the potential solo-LTRs, to further distinguish the solo-LTRs from the fragmented LTR retroelements and obtain the high-quality solo-LTRs for downstream analysis. Utilizing a word size of 4 in BLAST, we allowed the common length of 4 to 6 bp target site duplications (TSDs) for LTR retrotransposons as reported in Wicker et al.[1], and followed the approach outlined in Peona et al.[2]. Considering the removal process of transposons would lead to the target site duplications (TSDs) being imperfect and generating sequence diversity[3], it might cause the specific boundaries of TSDs and LTRs to not be so clear[4]. To address this, we not only scaned the TSDs of the 10 bp upstream and downstream of LTRs as mentioned in Peona et al.[2], but also extended the flanking region into 15 bp and 20 bp.

Overall, the evolutionary patterns of solo-LTRs were consistent (e.g. the high solo-LTR formations in birds and the ever-accelerating expansion in the Passeriformes group) using either all potential solo-LTRs or solo-LTRs with TSDs. The proportions of solo-LTRs relative to genome sizes in birds were still significantly lower than those for mammals under different flanking TSDs criteria (eg. TSDs in the 20bp flanking region, Welch's t-test, *p-value* = 0.0001; inner circle in **Supplementary Fig. 11** and **Supplementary Data 8**), but not significantly lower than those for reptiles (eg. TSDs in the 20bp flanking region, Welch's t-test, *p-value* = 0.2820; inner circle in **Supplementary Fig. 11** and **Supplementary Data 8**). While the significant positive correlations between the genome size and the number of solo-LTRs with TSDs were still observed in the reptile and mammal species, the bird species did not show this pattern (eg. TSDs in the 20bp flanking region, **Supplementary Fig. 12**). Moreover, we still found that birds had significantly higher frequencies of solo-LTRs formation in comparison with reptiles and mammals (eg. TSDs in the 20bp flanking region, Welch's t-test, *p-value* = 0.0001 and 0.0024, respectively; outer circle in **Supplementary Fig. 11** and **Supplementary Data 8**; more detailed statistical results of other flanking TSDs criteria were listed in **Supplementary Data 8**). ERVK solo-LTRs still showed similar patterns of ever-accelerating expansions in the Passeriformes group at different flanking TSDs criteria (**Supplementary Fig. 13** and **Supplementary Fig. 14**). Such continually spreading in the genomes of Passeriformes was accompanied with their diversification process in this group, especially in the crown Passerida (**Supplementary Fig. 14** and **Supplementary Data 9**).

For the insertion history of ERVK solo-LTRs, the conclusions of different flanking TSDs criteria were also consistent (**Supplementary Fig. 15**). Taking solo-LTRs with TSDs in the 20bp flanking region as an example, we found that an average of 45.34% ERVK solo-LTRs were species-specific in the suborder Passeri, and ERVK solo-LTRs shared between any two Passeri species under

the parvorder Passerida made up a large proportion (an average of 77.32%) among all ERVK solo-LTRs of their genomes. However, only a few shared ERVK solo-LTRs which could be traced to the common ancestral nodes of Passeri, Passerides, and Passerida (eg. TSDs in the 20bp flanking region, on average of 8.32%, 0.84%, and 2.12%, respectively). What's more, the signal of recent population diversity in ERVK solo-LTRs with TSDs remained. To be specific, 2.57% of ERVK solo-LTRs with TSDs in the 20bp flanking region were polymorphic among 19 zebra finch individuals based on the whole genome re-sequencing data.

Supplementary Figures

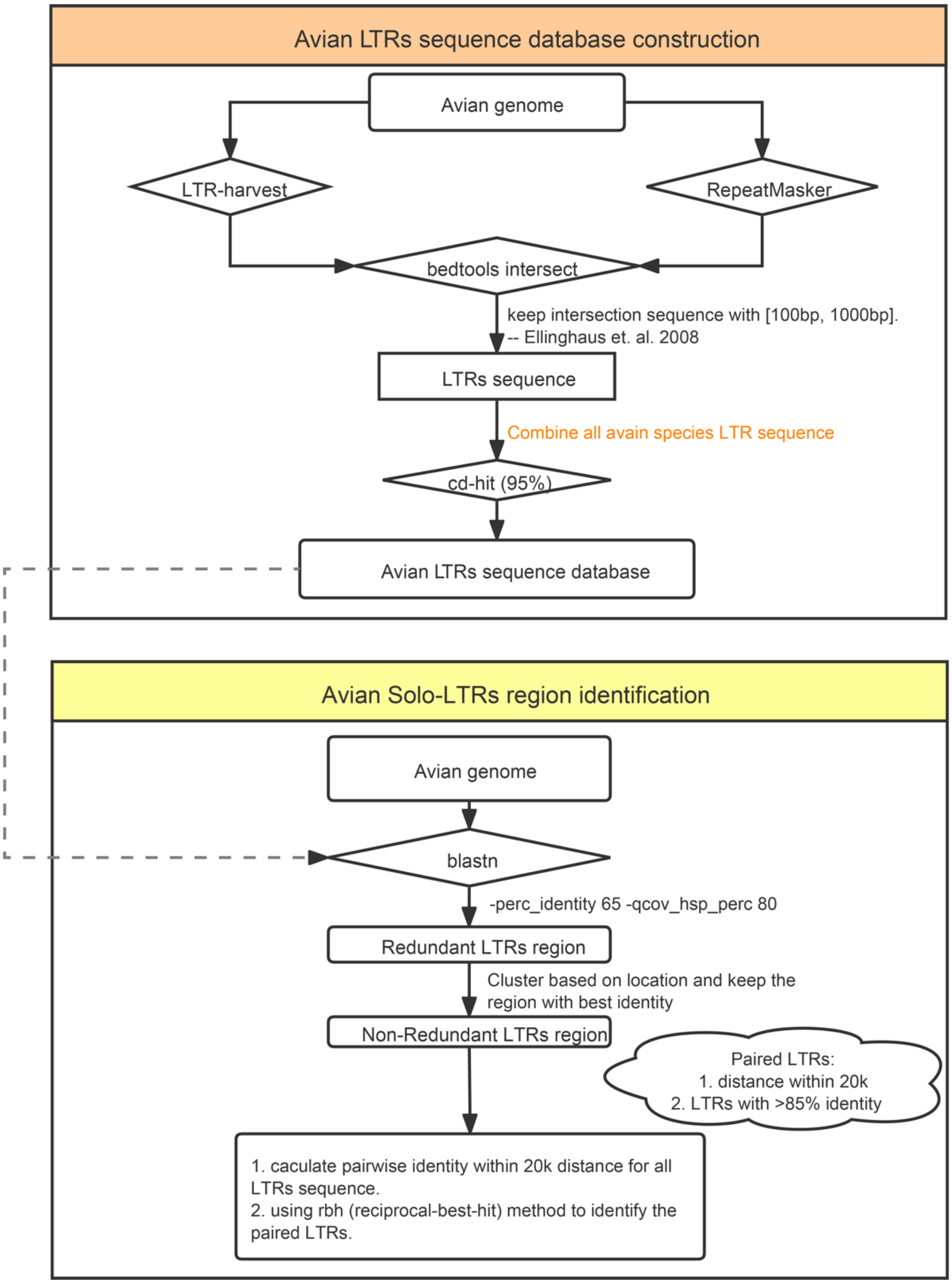

Supplementary Fig. 1: Pipeline for the identification of solo-LTRs in bird genomes.

Step 1: Construction of the LTRs sequence database for birds. Step 2: Identification of solo-LTRs regions in bird genomes.

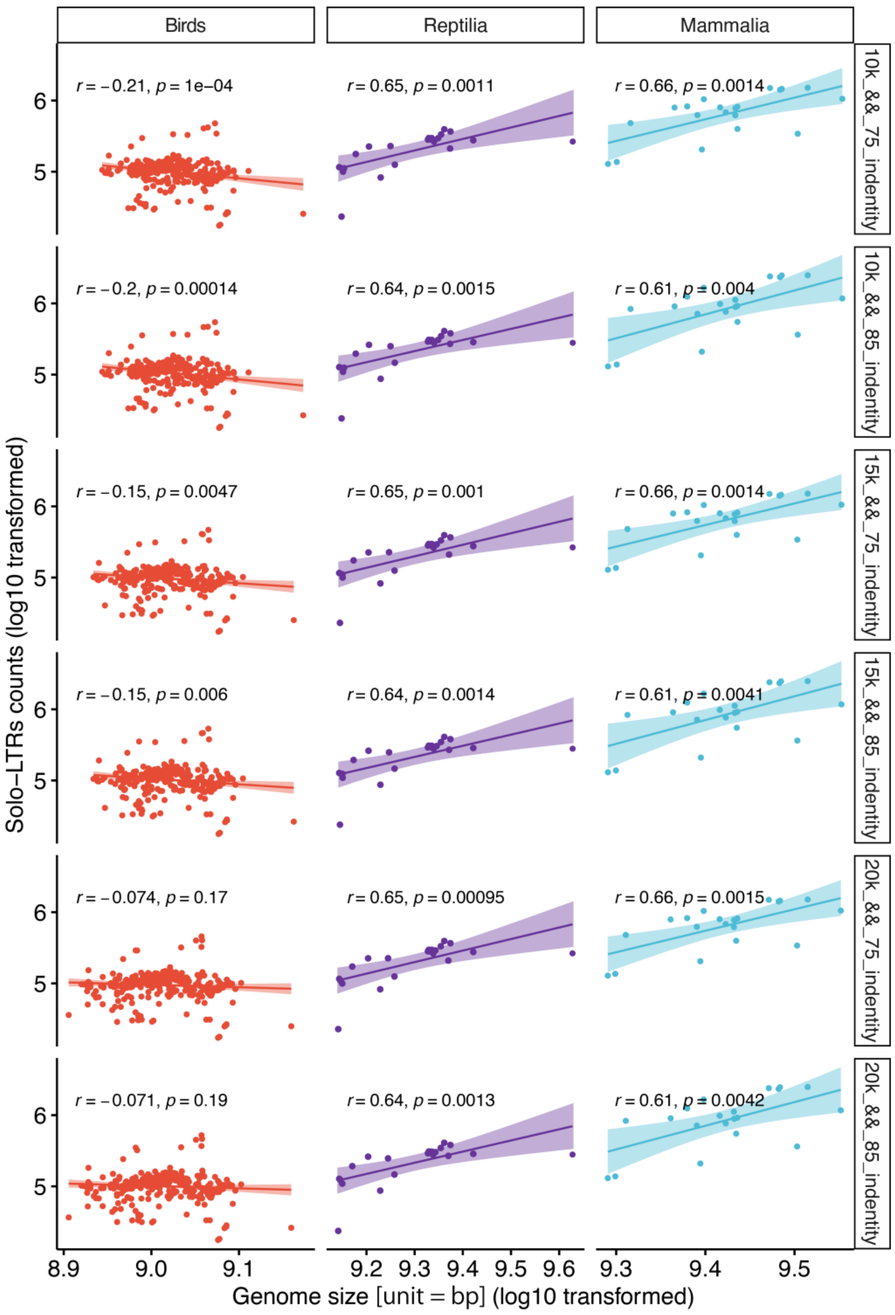

Supplementary Fig. 2: Solo-LTRs counts negatively correlate with genome sizes in bird species at various thresholds.

Bird and reptile species with potentially problematic assemblies (genome size < 800Mb or scaffold N50 < 20kb) were filtered to reduce potential bias from assembly quality. Dots correspond to individual species, with red dots indicating bird species (n=345), purple dots indicating reptile species (n=22), and bule dots indicating mammal species (n=20). Colored regions indicate the 95% confidence interval for each regression line. Source data are provided as a Source Data file.

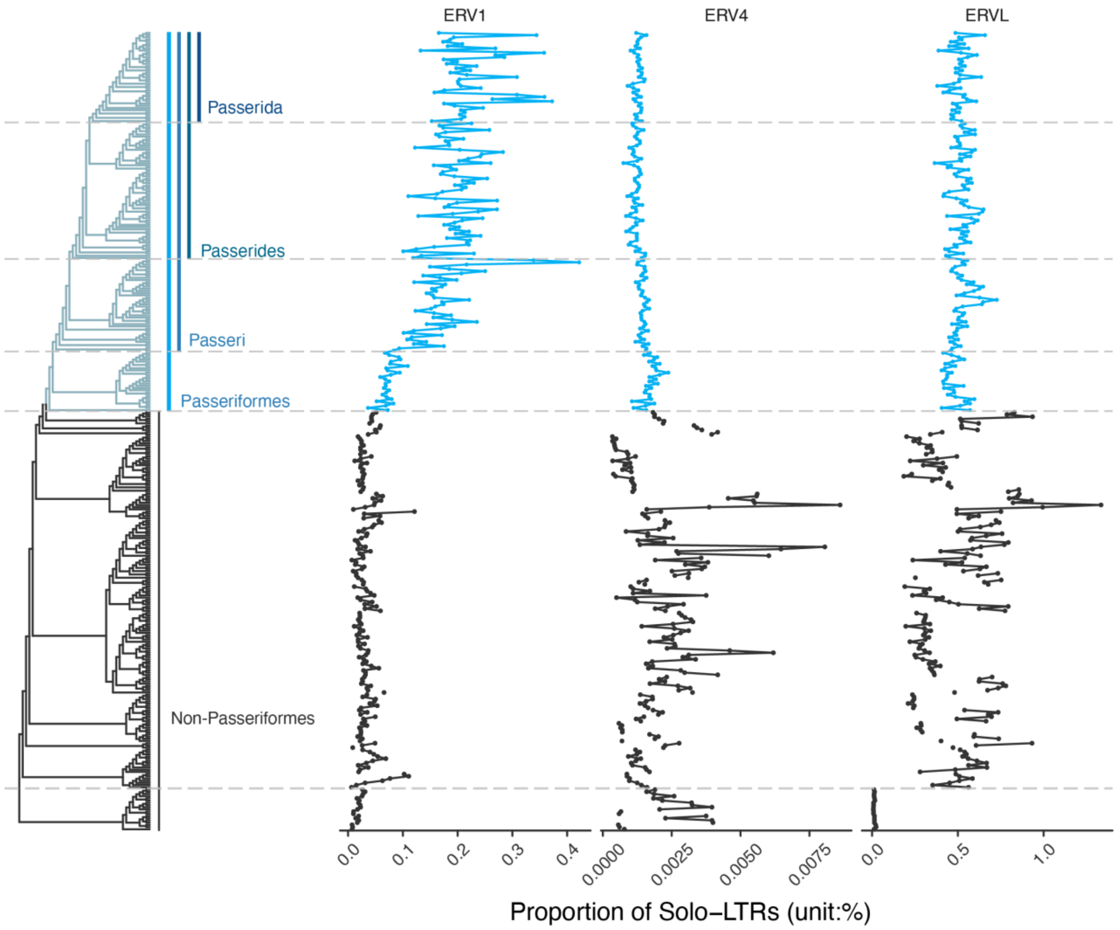

Supplementary Fig. 3: Proportions of other three types of ERV solo-LTRs mapped on the phylogenetic lineages of bird species.

Bule dots indicate Passeriformes species (n=172), and black dots indicate Non-Passeriformes species (n=190). The pattern of ERV1 solo-LTRs was more likely to be explained as a concentrated expansion at an ancestral node of Passeriformes, rather than a continual spreading in the genomes accompanying with the Passeriformes diversification process shown by ERVK solo-LTRs in **Fig. 3a**.

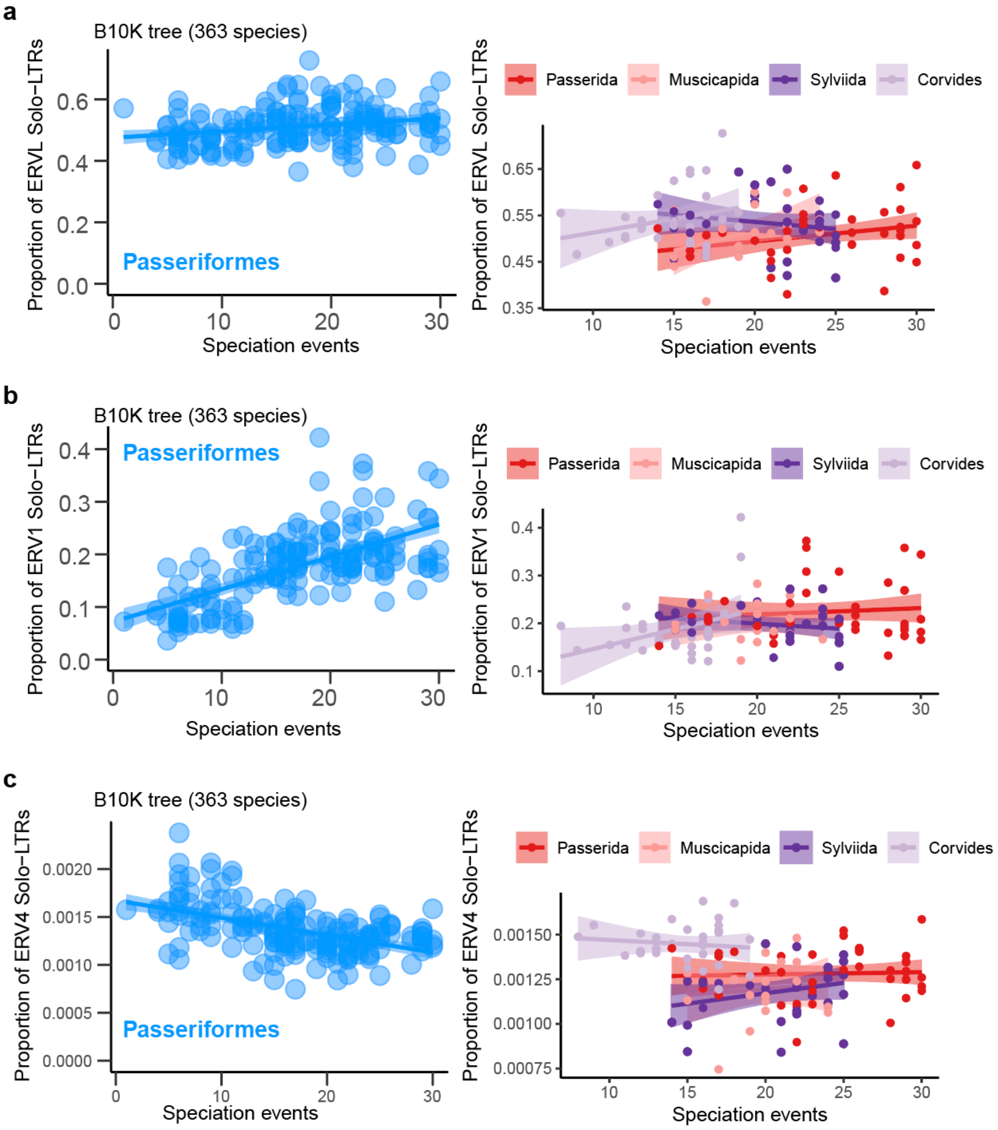

Supplementary Fig. 4: Other ERV solo-LTRs in Passeriformes bird species.

a, ERVL, **b**, ERV1, and **c**, ERV4 solo-LTRs do not show increased accumulations with speciation events in the parvorder Passerida in contrast with ERVK.Speciation events were measured as the number of nodes along a path from the Passeriformes ancestor node to the tips of each species, based on B10K family-level bird phylogeny. Each dot represents a species and each color of dots represents a clade of birds (n=169, 41, 20, 33, and 30 for Passeriformes, Passerida, Muscicapida, Sylviida and Corvides, respectively). Colored regions indicate the 95% confidence interval for each regression line. Details of the Pearson's correlation tests (including *p-value* and Pearson's r) and the linear regression analyses were listed in **Supplementary Data 2**. Source data are provided as a Source Data file.

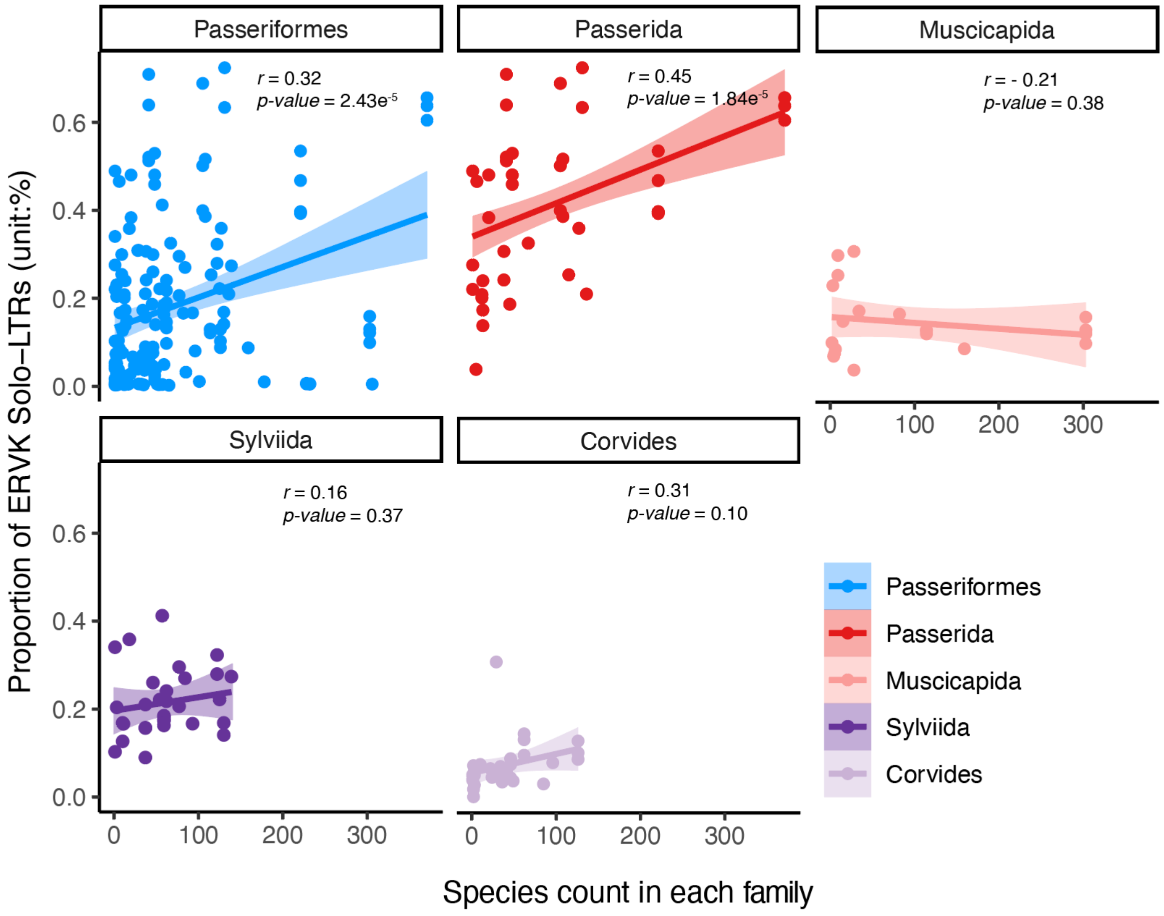

Supplementary Fig. 5: The accumulation of ERVK solo-LTRs in Passeriformes species based on the phylogenetic tree of the 10,135 bird species.

Using the species counts in each family from the phylogenetic relationship of the extant 10,135 bird species to avoid potential sampling bias, the accelerated pattern of accumulation of ERVK solo-LTRs still persists in the parvorder Passerida. Each dot represents a species and each color of dots represents a clade of birds (n=169, 41, 20, 33, and 30 for Passeriformes, Passerida, Muscicapida, Sylviida and Corvides, respectively). Colored regions indicate the 95% confidence interval for each regression line. Details of the Pearson's correlation tests (including *p-value* and Pearson's r) and the linear regression analyses were listed in **Supplementary Data 2**. Source data are provided as a Source Data file.

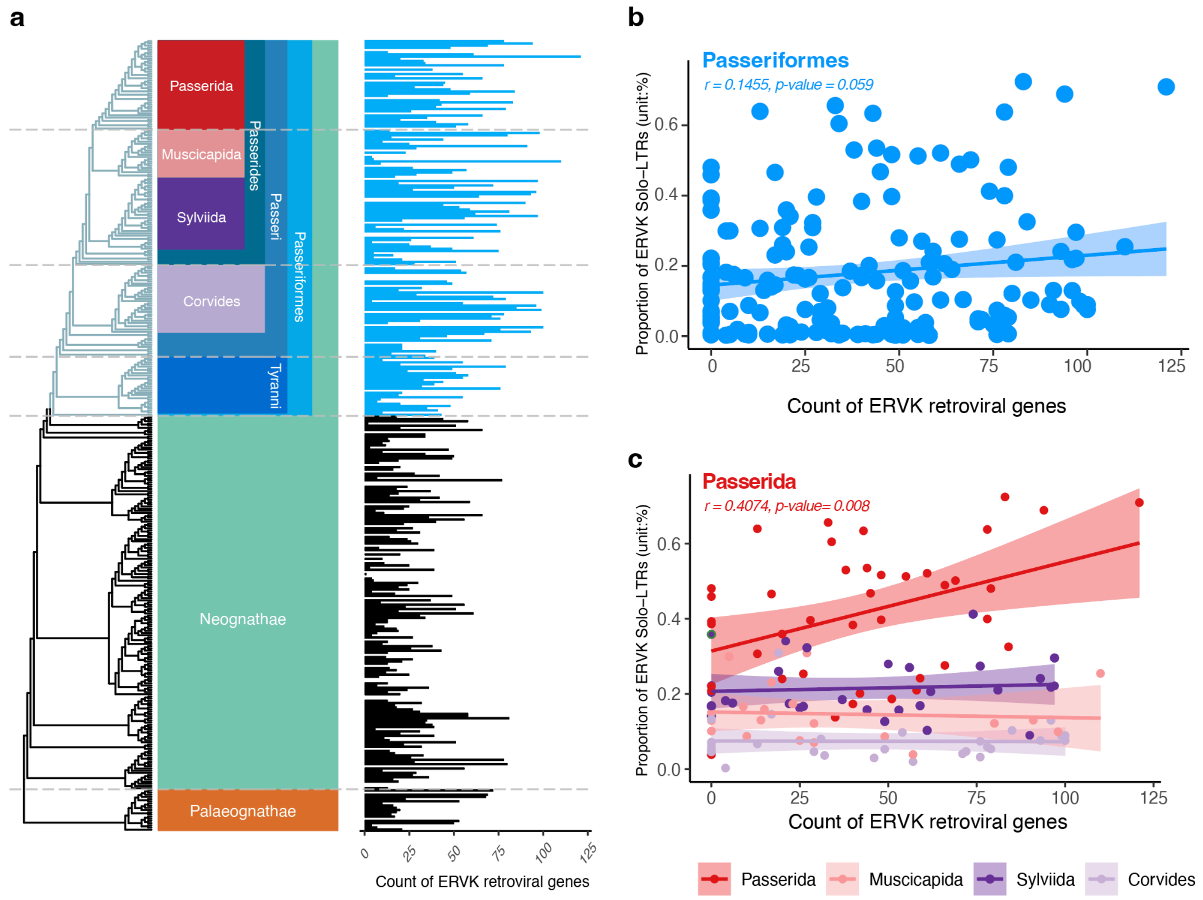

Supplementary Fig. 6: ERVK viral genes showed a similar accelerated pattern of accumulation with the proportion of ERVK solo-LTRs in the parvorder Passerida.

a, The phylogenetic distribution of ERVK viral genes across species of Passeriformes. Bule bars indicate Passeriformes species (n=172), and black bars indicate Non-Passeriformes species (n=190). **b,** ERVK solo-LTRs didn't correlate with proportion of ERVK viral genes in genomes of Passeriformes species, but positively correlated in **c,** the parvorder Passerida. Colored regions indicate the 95% confidence interval for each regression line (n=169, 41, 20, 33, and 30 for Passeriformes, Passerida, Muscicapida, Sylviida and Corvides, respectively). Source data are provided as a Source Data file.

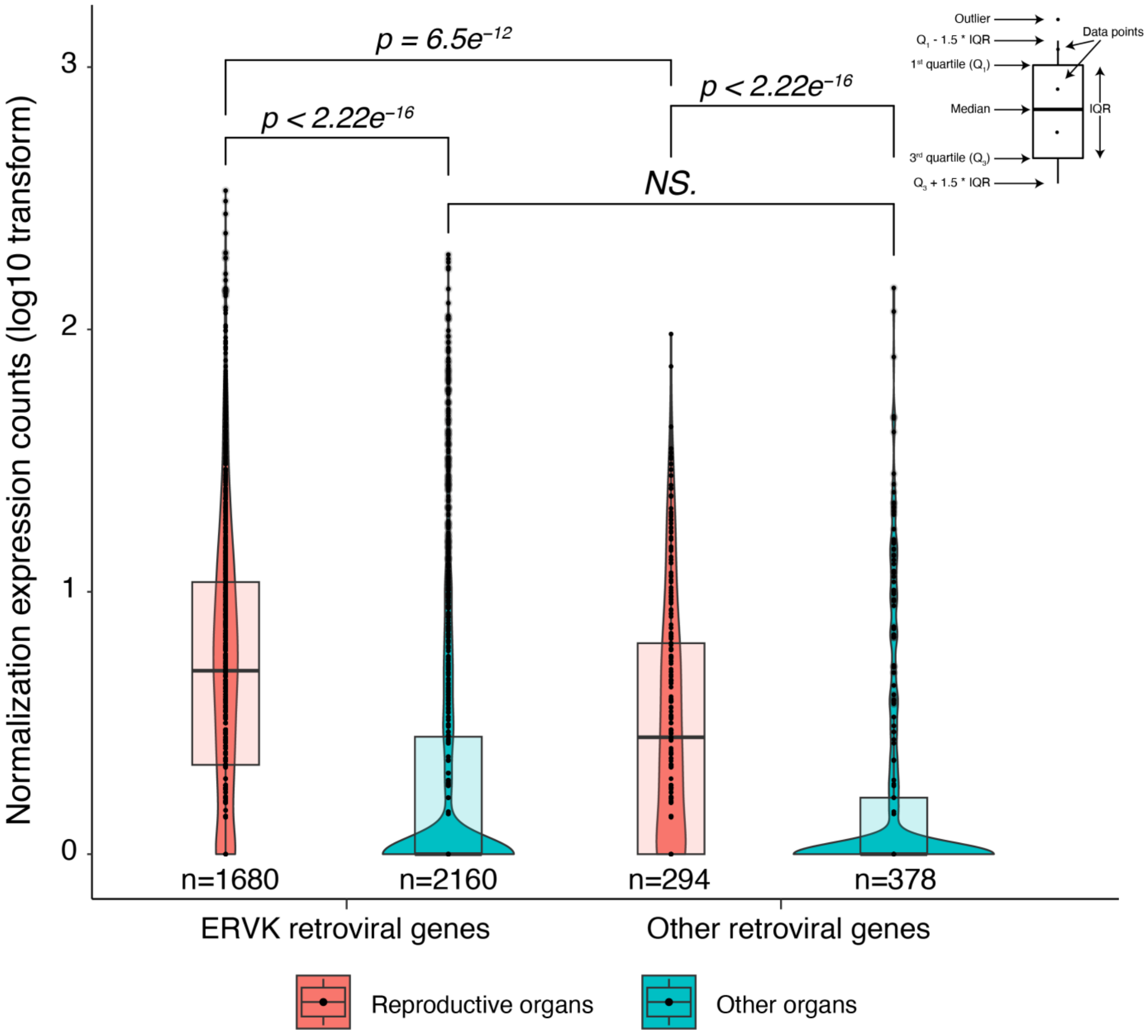

Supplementary Fig. 7: In zebra finches' reproductive organs, ERVK retroviral genes display significantly higher expression levels than other retroviral genes.

We applied a log10(count + 1) transformation to the normalized count for visualization. Welch's one-sided t-test was performed under one-sided. Sample size and definition of dots and lines of box plots could be found in legend. Source data are provided as a Source Data file.

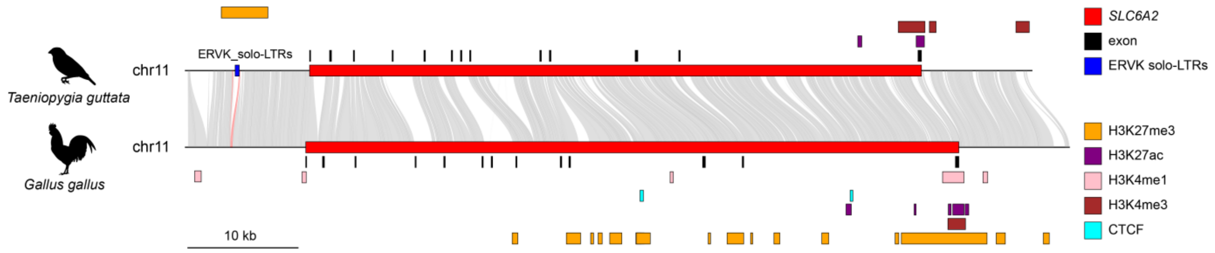

Supplementary Fig. 8: ERVK solo-LTRs contribute to the regulatory element region of the *SLC6A2* gene.

Silhouettes of the zebra finch and chicken are from https://www.phylopic.org/.

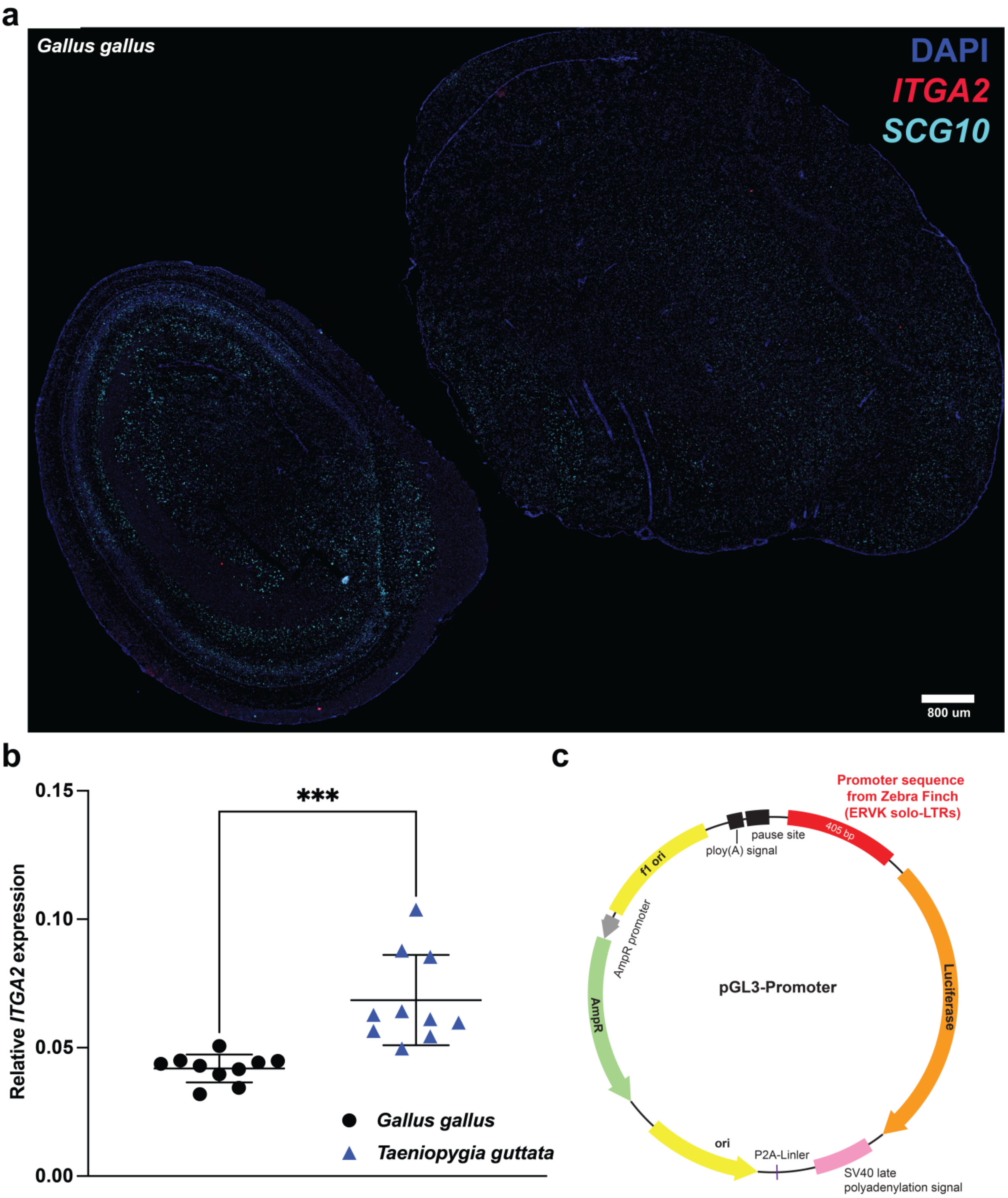

Supplementary Fig. 9: Results of the FISH experiment and a plasmid map of the dual-luciferase reporter assay experiment.

a, Fluorescence *in situ* hybridization (FISH) microscope photographs of chicken's brain. The expression signals of *ITGA2* and *SCG10* were detected and highlighted in red and cyan, respectively. The blue signal is from 4′ 6-diamidino-2-phenylindole (DAPI) staining, showing the location of the nucleus. **b**, Relative *ITGA2* expression to the *SCG10* signal revealed the higher expression of this gene in the zebra

finch than in the chicken. The *ITGA2* and *SCG10* signals were measured using the “Analytical Particle” function of the software FIJI with ten random samples (n=10) taken across the whole brain. Horizontal lines indicate the mean (± s.d.). The Welch’s two-tailed t-test was performed using GraphPad Prism software, with the *p-value*=0.0009 (*** <0.001). Source data are provided as a Source Data file. **c**, The plasmid map of the pGL3-Promoter vector.

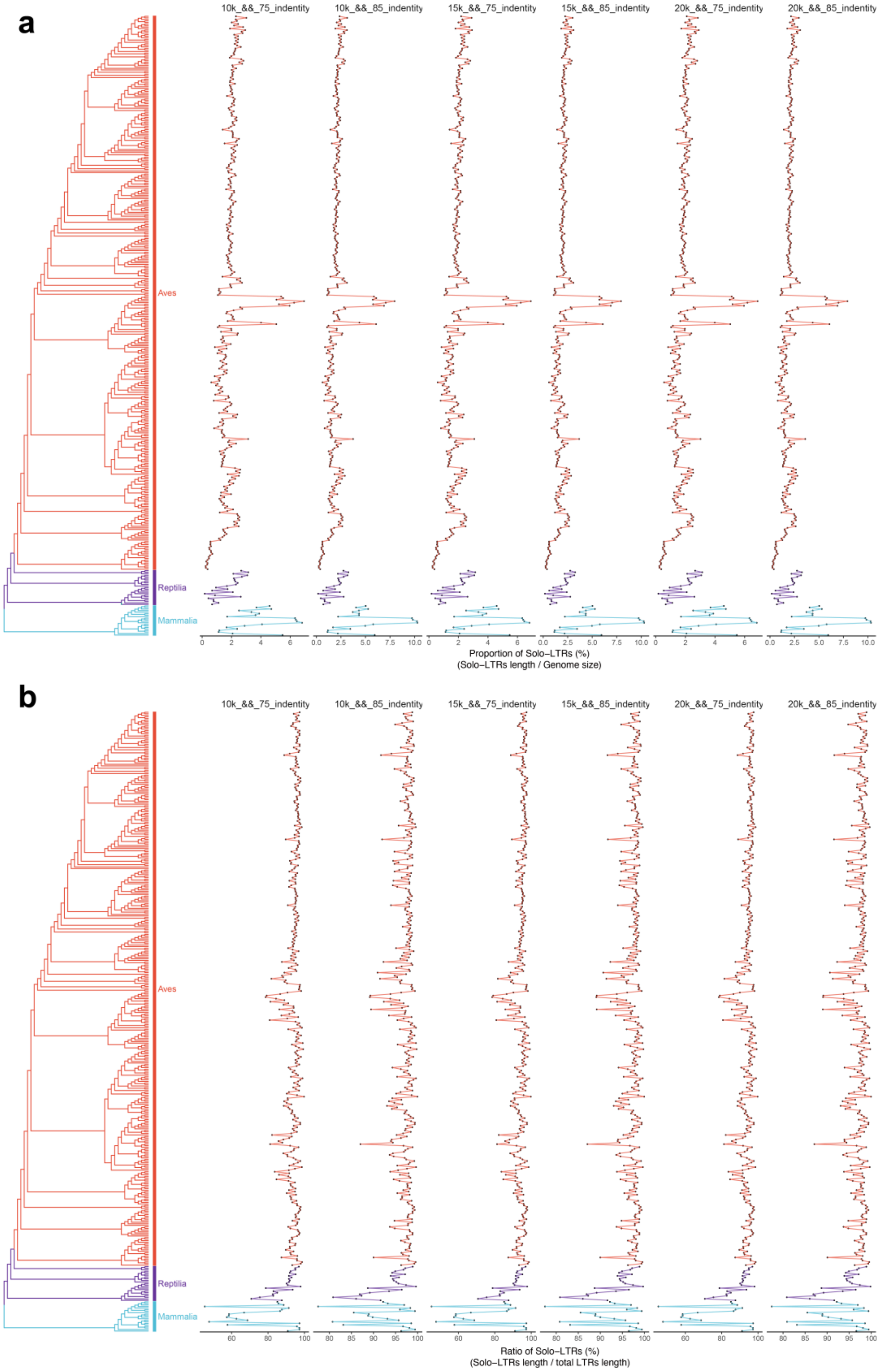

Supplementary Fig. 10: Different thresholds used in the identification of solo-LTRs.

a, The proportion of solo-LTRs, and **b,** the ratio of solo-LTRs formation among birds, reptiles, and mammals. Dots correspond to individual species, with red dots indicating bird species (n=362), purple dots indicating reptile species (n=23), and blue dots indicating mammal species (n=20).

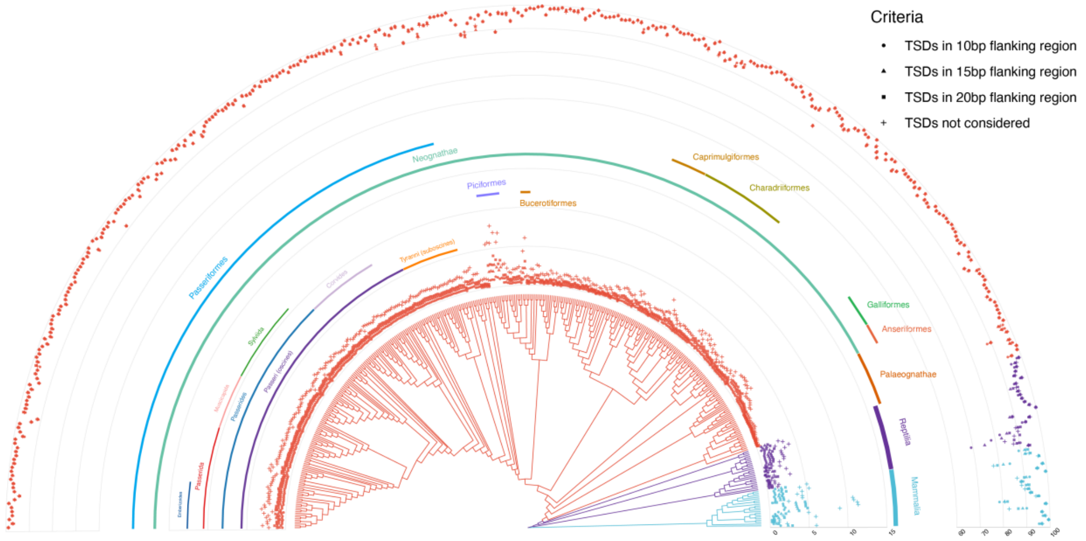

Supplementary Fig. 11: Phylogenetic tree with the proportion of solo-LTRs (the inner circle) and the ratio of solo-LTR (outer circle) in birds, reptiles, and mammals under different TSDs criteria. Dots correspond to individual species, with red dots indicating bird species (n=362), purple dots indicating reptile species (n=23), and blue dots indicating mammal species (n=20). The inner circle represents the proportion of solo-LTRs relative to the genome size. The outer circle represents the ratio of solo-LTRs to total LTRs length, indicating the frequency of solo-LTRs formation. The shape of the points represents the different criteria for searching the TSDs in the flanking region of the solo-LTRs. Taxonomic information follows classifications in Howard and Moore.

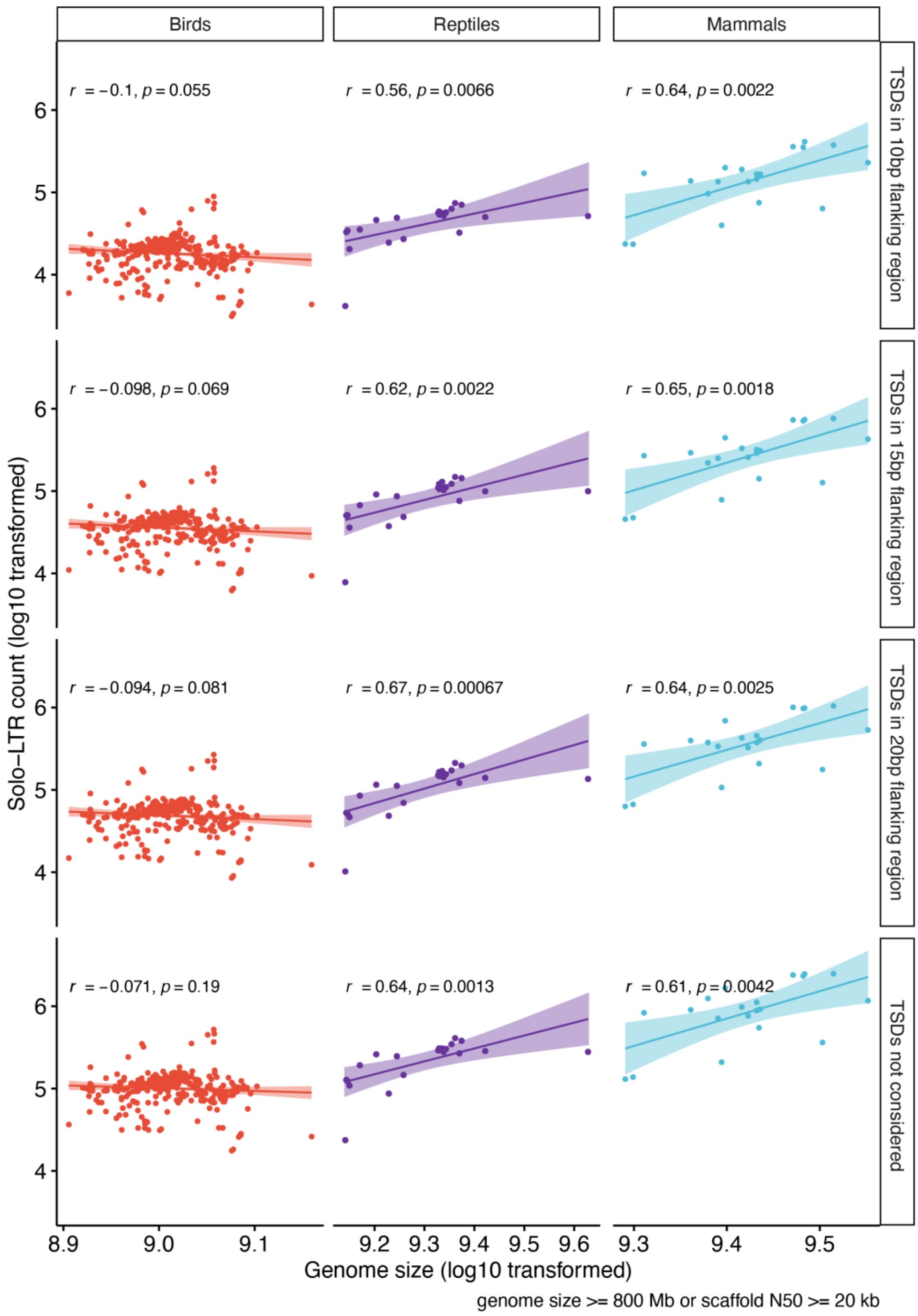

Supplementary Fig. 12: Solo-LTRs counts were positively correlated with the genome size in mammals and reptiles, but not in birds under different TSDs criteria.

Bird and reptile species with potentially problematic assemblies (genome size < 800 Mb or scaffold N50 < 20 kb) were filtered to reduce the bias of assembly quality. Dots correspond to individual species, with red dots indicating bird species (n=345), purple dots indicating reptile species (n=22), and bule dots indicating mammal species (n=20). Colored regions indicate the 95% confidence interval for each regression line. Source data are provided as a Source Data file.

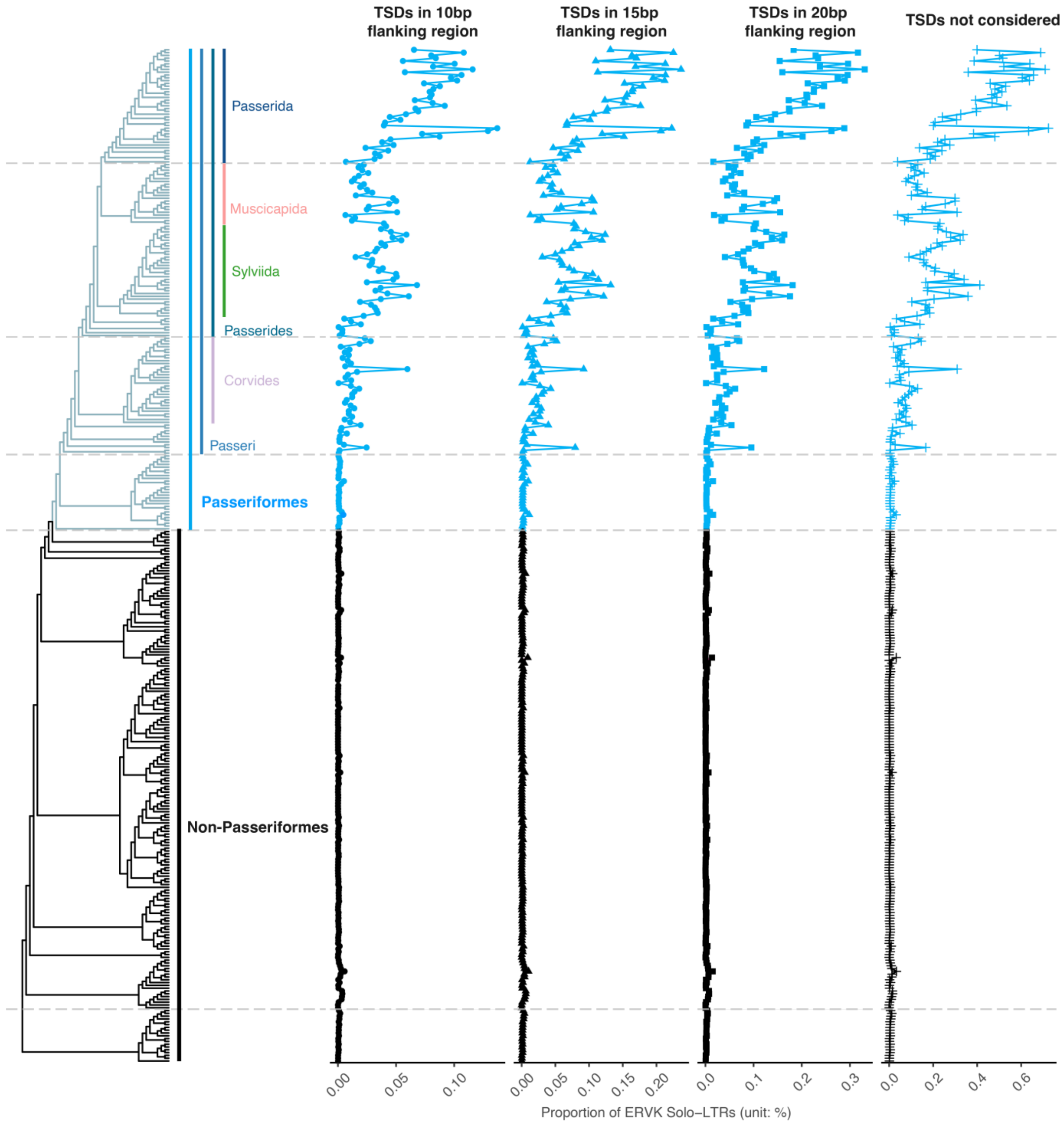

Supplementary Fig. 13: ERVK solo-LTRs accumulated in Passeriformes under different TSDs criteria.

Phylogenetic tree illustrating the proportion of ERVK solo-LTRs in bird species. Bule dots indicate Passeriformes species (n=172), and black dots indicate Non-Passeriformes species (n=190). Branches correspond to different species; from top to bottom, the branches above the five gray dashed lines correspond to species in Passerida, Passerides, Passeri, Passeriformes, and Neognathae, respectively. Based on the B10K family-level bird phylogeny.

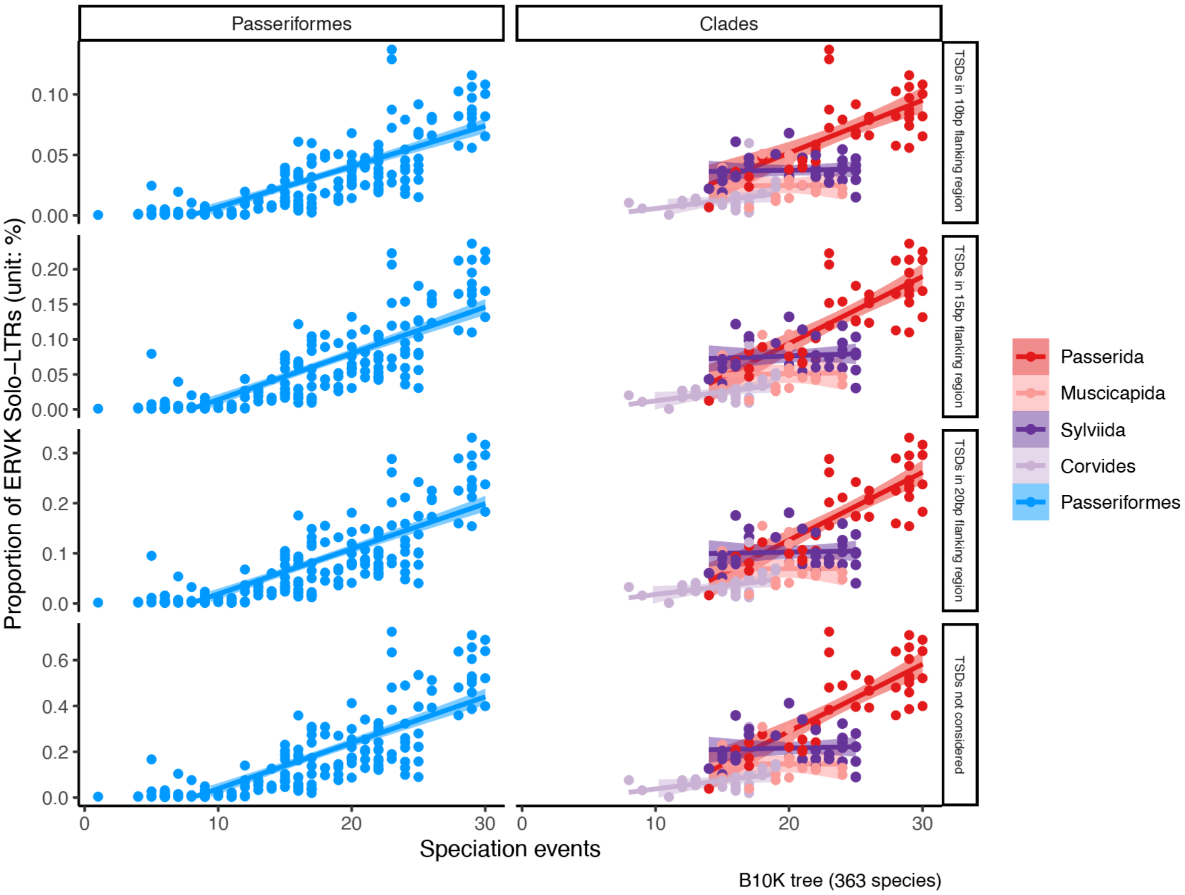

Supplementary Fig. 14: ERVK solo-LTRs accumulate during speciation events in Passeriformes, especially in Passerida, under different TSDs criteria.

Speciation events were measured as the number of nodes along a path from the Passeriformes ancestor node to the tips of each species, based on B10K family-level bird phylogeny. Each dot represents a species and each color of dots represents a clade of birds (n=169, 41, 20, 33, and 30 for Passeriformes, Passerida, Muscicapida, Sylviida and Corvides, respectively). Details of the Pearson's correlation tests (including *p-value* and Pearson's r) and the linear regression analyses were listed in **Supplementary Data 9**. Colored regions indicate the 95% confidence interval for each regression line. Source data are provided as a Source Data file.

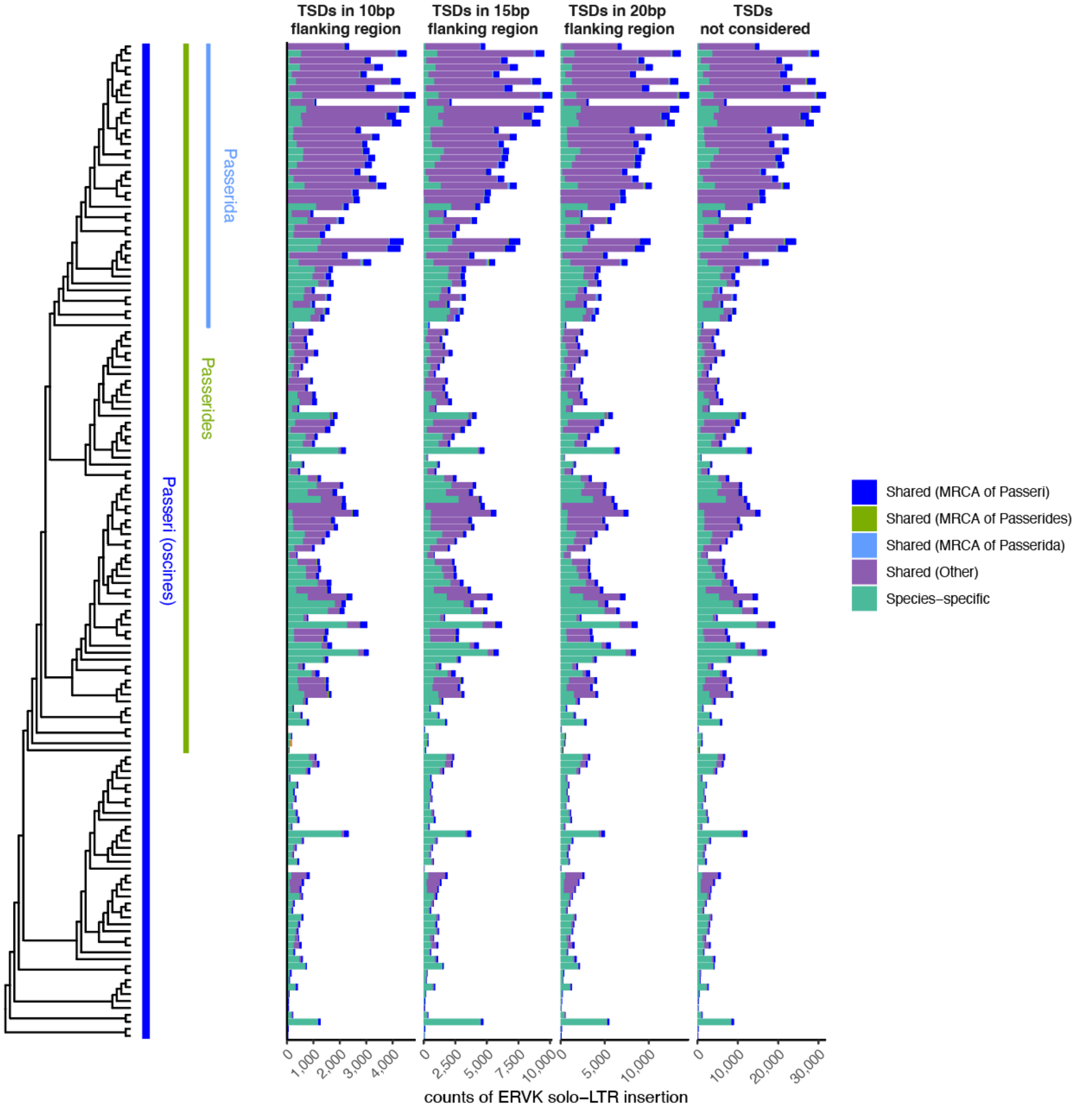

Supplementary Fig. 15: Shared ERVK solo-LTRs constitute a large portion of all ERVK solo-LTRs in Passerida bird species under different TSDs Criteria.

The sample size for Passeri, Passerides and Passerida bird species are n=143, 101 and 41, respectively. Source data are provided as a Source Data file.

Supplementary Reference

1 Wicker, T. *et al.* A unified classification system for eukaryotic transposable elements. *Nat. Rev. Genet.* **8**, 973-982, doi:10.1038/nrg2165 (2007).
2 Peona, V. *et al.* The hidden structural variability in avian genomes. *bioRxiv*, 2021.2012.2031.473444, doi:10.1101/2021.12.31.473444 (2022).
3 Fedoroff, N. V. Transposable Elements, Epigenetics, and Genome Evolution. *Science* **338**, 758-767, doi:doi:10.1126/science.338.6108.758 (2012).
4 Ji, Y. & DeWoody, J. A. Genomic Landscape of Long Terminal Repeat Retrotransposons (LTR-RTs) and Solo LTRs as Shaped by Ectopic Recombination in Chicken and Zebra Finch. *Journal of Molecular Evolution* **82**, 251-263, doi:10.1007/s00239-016-9741-0 (2016).
