# Supplementary material for: Adaptive expansion of ERVK solo-LTRs is associated with Passeriformes speciation events
Source: Nat Commun. 2024 Apr 11;15:3151. doi: 10.1038/s41467-024-47501-3 (PMC11009239; doi:10.1038/s41467-024-47501-3)
Supplement: Supplementary file 3 — Description of Additional Supplementary Files [file 41467_2024_47501_MOESM3_ESM.pdf]

Description of Additional Supplementary Files

Supplementary Data 1

Description: Species information of 362 aves, 23 reptiles, and 20 mammals

Supplementary Data 2

Description: Results of of Pearson's correlation tests under 95% confidence interval for different clades and different types of soloLTRs

Supplementary Data 3

Description: Data used in Pearson's correlation tests for different clades and different types of solo-LTR

Supplementary Data 4

Description: Data repository of RNAseq data, population re-sequencing data, and ChIP-seq data of zebra finchs and chickens used in the study

Supplementary Data 5

Description: Evaluation of the significance of enrichment ERVK solo-LTRs with ChIP-seq peaks with Fisher's one-sided exact test

Supplementary Data 6

Description: GO enrichment results of genes with the regulatory elements contributed by ERVK soloLTRs

Supplementary Data 7

Description: Statistics of threshold test results with different filtering criteria

Supplementary Data 8

Description: Results of statistical tests and covariance analyses under different TSDs criteria

Supplementary Data 9

Description: Results of Pearson's correlation tests between the proportion of Solo-LTRs and the speciation events under different TSDs criteria in different clades
