# Supplementary material for: Adaptive expansion of ERVK solo-LTRs is associated with Passeriformes speciation events
Source: Nat Commun. 2024 Apr 11;15:3151. doi: 10.1038/s41467-024-47501-3 (PMC11009239; doi:10.1038/s41467-024-47501-3)
Supplement: Supplementary file 13 — Reporting Summary [file 41467_2024_47501_MOESM13_ESM.pdf]

Corresponding author(s): Shaohong Feng
Last updated by author(s): 2024/03/13

Reporting Summary

Nature Portfolio wishes to improve the reproducibility of the work that we publish. This form provides structure for consistency and transparency in reporting. For further information on Nature Portfolio policies, see our Editorial Policies and the Editorial Policy Checklist.

Statistics

For all statistical analyses, confirm that the following items are present in the figure legend, table legend, main text, or Methods section.

n/a	Confirmed	
☐	☒	The exact sample size (*n*) for each experimental group/condition, given as a discrete number and unit of measurement
☐	☒	A statement on whether measurements were taken from distinct samples or whether the same sample was measured repeatedly
☐	☒	The statistical test(s) used AND whether they are one- or two-sided<br>*Only common tests should be described solely by name; describe more complex techniques in the Methods section.*
☒	☐	A description of all covariates tested
☐	☒	A description of any assumptions or corrections, such as tests of normality and adjustment for multiple comparisons
☐	☒	A full description of the statistical parameters including central tendency (e.g. means) or other basic estimates (e.g. regression coefficient) AND variation (e.g. standard deviation) or associated estimates of uncertainty (e.g. confidence intervals)
☐	☒	For null hypothesis testing, the test statistic (e.g. *F*, *t*, *r*) with confidence intervals, effect sizes, degrees of freedom and *P* value noted<br>*Give P values as exact values whenever suitable.*
☒	☐	For Bayesian analysis, information on the choice of priors and Markov chain Monte Carlo settings
☒	☐	For hierarchical and complex designs, identification of the appropriate level for tests and full reporting of outcomes
☐	☒	Estimates of effect sizes (e.g. Cohen's *d*, Pearson's *r*), indicating how they were calculated

Our web collection on statistics for biologists contains articles on many of the points above.

Software and code

Policy information about availability of computer code

Data collection	The brains section were cryosectioned in the sagittal plane at 10 μm using a freezing microtome machine (Leica CM1950, Germany). The images were acquired with a Leica DM6B fluorescence microscope (Leica, Germany) using a 20 × objective and exported in TIFF format, and further image processing about regions of interest (ROIs) was conducted using FIJI (https://imagej.net/Fiji). Chicken fibroblast cell lines (UMNSAH/DF-1) were maintained at 38°C in an incubator with a humidified atmosphere of 5% CO_2, and prepared in 12-well plates. The pGL3-Promoter and pRL-TK vector (Promega) were co-transfected to cells using Lipofectamine 3000 reagent (Invitrogen, USA) (in accordance with the manufacturer's instructions), with pGL3-Basic empty vector as a control. The luciferase reporter assay was conducted using the Dual-Luciferase Reporter Assay System (E1910, Promega, USA). Firefly and Renilla luciferase activities were measured using a GloMax 96 Microplate Luminometer (Promega, USA).
Data analysis	All open source code and custom code used in this study Code has been deposited in GitHub (https://github.com/ChenGuangji/BirdsSoloLTRs) and Zenodo (https://doi.org/10.5281/zenodo.10812365), including R package ggtree v3.3.0.900, R package UpSetR v.1.4.0, R package AnnotationForge v.1.42.0, RepeatMasker v4.1.2, RepBase library v.20170127, LTR-harvest v1.6.1, bedtools v2.30.0, blastn v2.9.0, KneadData v0.10.0, SILVA ribosomal RNA database v0.2, Hisat2 v.2.2.1, HTSeq package v.0.13.5, DESeq2 v.1.34.0, eggNOG-mapper, hal2maf, REVIGO tool, FIJI, FindM tool, BWA-MEM, .

For manuscripts utilizing custom algorithms or software that are central to the research but not yet described in published literature, software must be made available to editors and reviewers. We strongly encourage code deposition in a community repository (e.g. GitHub). See the Nature Portfolio guidelines for submitting code & software for further information.

Data

Policy information about availability of data

All manuscripts must include a data availability statement. This statement should provide the following information, where applicable:

- Accession codes, unique identifiers, or web links for publicly available datasets
- A description of any restrictions on data availability
- For clinical datasets or third party data, please ensure that the statement adheres to our policy

The 362 avian genome assemblies and annotations data are accessible through the CNSA public database (https://db.cngb.org/cnsa/) with accession number CNP0000505 and NCBI database with accession PRJNA545868. The other 42 genome assemblies, DNA sequencing and RNA-seq data used in this study can be found in the NCBI public database according to Supplementary Data 1 and Supplementary Data 4. Source data are provided as Source Data files and availabled on Zenodo (https://doi.org/10.5281/zenodo.10812365).

Research involving human participants, their data, or biological material

Policy information about studies with human participants or human data. See also policy information about sex, gender (identity/presentation), and sexual orientation and race, ethnicity and racism.

Reporting on sex and gender	The study does not involve human participants or human data.
Reporting on race, ethnicity, or other socially relevant groupings	The study does not involve human participants or human data.
Population characteristics	The study does not involve human participants or human data.
Recruitment	The study does not involve human participants or human data.
Ethics oversight	The study does not involve human participants or human data.

Note that full information on the approval of the study protocol must also be provided in the manuscript.

Field-specific reporting

Please select the one below that is the best fit for your research. If you are not sure, read the appropriate sections before making your selection.

☐ Life sciences ☐ Behavioural & social sciences ☒ Ecological, evolutionary & environmental sciences

For a reference copy of the document with all sections, see nature.com/documents/nr-reporting-summary-flat.pdf

Ecological, evolutionary & environmental sciences study design

All studies must disclose on these points even when the disclosure is negative.

Study description	The study investigates solo-LTRs formation among bird, reptiles and mammals species, reveals an unusually higher level of solo-LTRs formation in birds, indicating evolutionary forces might have purged ERVs during evolution. This study used fluorescence in situ hybridization to reveal the differential expression of ITGA2 in brain region of zebra finches (Taeniopygia guttata) and chickens (Gallus gallus). This study also used a dual-luciferase reporter assay to verify the potential cis-regulatory of recently evolved ERVK solo-LTRs in zebra finches.
Research sample	We identified solo-LTRs from the genomes of 405 published representative amniote species (including 362 birds, 23 reptiles, and 20 mammals). We conducted fluorescence in situ hybridization experiments on zebra finches (Taeniopygia guttata, age 2–3 months, represented Passeriformes bird species) and chickens (Gallus gallus, age 2–3 months, represented Non-Passeriformes bird species), to reveal the differential expression of ITGA2 in brain region. We verified the potential cis-regulatory of recently evolved ERVK solo-LTRs in chicken fibroblast cell line (UMNSAH/DF-1) via the dual-luciferase reporter assay experiments.
Sampling strategy	We targeted on the published representative amniote species from the public database(NCBI), including 362 birds, 23 reptiles, and 20 mammals. The brains section of zebra finches and chickens were cryosectioned in the sagittal plane at 10 µm using a freezing microtome machine (Leica CM1950, Germany). The target brain region section were sampling between 2.00 and 2.25 mm lateral to the midline for zebra finch and between 3.40 and 3.90 mm lateral to the midline for chicken. The luciferase activity of dual-luciferase reporter assay was using a GloMax 96 Microplate Luminometer (Promega, USA) with n=9 biologically independent repetitions for each experimental group.
Data collection	We identified solo-LTRs and paired-LTRs loci from all species's genome assembly using bioinformatic methods. Firefly and Renilla luciferase activities were measured using a GloMax 96 Microplate Luminometer (Promega, USA), operated by Guangji Chen and Dan Yu. The fluorescence in situ hybridization images were acquired with a Leica DM6B fluorescence microscope (Leica, Germany),

	operated by Yu Yang. The image processing about regions of interest (ROIs) was conducted using FIJI (https://imagej.net/Fiji), operated by Guangji Chen.
Timing and spatial scale	The data of solo-LTR formation were identified from the all species's genome assembly at the same batch. The luciferase activities were measured for n=9 biologically independent repetitions for each experimental group at the same batch. The fluorescence in situ hybridization images were acquired with a Leica DM6B fluorescence microscope (Leica, Germany) at the same day. The image processing about regions of interest (ROIs) was conducted using FIJI (https://imagej.net/Fiji) at the same batch.
Data exclusions	We excluded the short scaffold (<20k) to minimize false positives for solo-LTR identification. We excluded the poor bird assembly (genome size <800M or scaffold N50 <20k) in the correlation analysis of absolute solo-LTRs count value with genome size, to reduce the impact of assembly quality.
Reproducibility	We performed the threshold test for solo-LTR identification and testing of the target site duplications, to support the consistent of evolutionary patterns of solo-LTRs formation in birds. The experiments were performed at least twice. In all cases, all attempts of replication were succesful.
Randomization	Decisions on identification were based on bioinformatic cutoffs, therefore randomization was not relevant.
Blinding	Decisions on identification were based on bioinformatic cutoffs, therefore blinding was not relevant.

Did the study involve field work? ☐ Yes ☒ No

Reporting for specific materials, systems and methods

We require information from authors about some types of materials, experimental systems and methods used in many studies. Here, indicate whether each material, system or method listed is relevant to your study. If you are not sure if a list item applies to your research, read the appropriate section before selecting a response.

Materials & experimental systems

n/a	Involved in the study	
☒	☐	Antibodies
☐	☒	Eukaryotic cell lines
☒	☐	Palaeontology and archaeology
☐	☒	Animals and other organisms
☒	☐	Clinical data
☒	☐	Dual use research of concern
☒	☐	Plants

Methods

n/a	Involved in the study	
☒	☐	ChIP-seq
☒	☐	Flow cytometry
☒	☐	MRI-based neuroimaging

Eukaryotic cell lines

Policy information about cell lines and Sex and Gender in Research

Cell line source(s)	The chicken fibroblast cell line (UMNSAH/DF-1) was originally purchased from Thermo Fisher Scientific and was maintained in our laboratory.
Authentication	The used cell line in this study is commercially available, and was authenticated by the manufacturer.
Mycoplasma contamination	All cells were tested for mycoplasma and proven negative.
Commonly misidentified lines (See ICLAC register)	No commonly misidentified cell lines were used.

Animals and other research organisms

Policy information about studies involving animals; ARRIVE guidelines recommended for reporting animal research, and Sex and Gender in Research

Laboratory animals	The zebra finches (Taeniopygia guttata, age 2–3 months) and chickens (Gallus gallus, age 2–3 months) used in the experiments were maintained in the Laboratory Animal Center and the Center for Evolutionary & Organismal Biology, Zhejiang University, Hangzhou, China
Wild animals	The study did not involve wild animals.
Reporting on sex	Sex was not considered in study design.
Field-collected samples	The study did not involve samples collected from the field for laboratory work.

Ethics oversight	All animal handling procedures were approved by the Animal Use and Care Committee of Zhejiang University following the Guidelines of the Care and Use of Laboratory Animals in China.

Note that full information on the approval of the study protocol must also be provided in the manuscript.
